# Supplementary material for: Overload wave-memory induces amnesia of a self-propelled particle
Source: Nat Commun. 2022 Jul 27;13:4357. doi: 10.1038/s41467-022-31736-z (PMC9329294; doi:10.1038/s41467-022-31736-z)
Supplement: Supplementary file 1 — Supplementary Information [file 41467_2022_31736_MOESM1_ESM.pdf]

Supplementary Information:
"Overload wave-memory induces amnesia of a self-propelled particle"

Maxime Hubert*
Friedrich-Alexander-Universität Erlangen-Nürnberg, Institute for Theoretical Physics, PULS Group, Interdisciplinary center for nanostructured films (IZNF), Cauerstr. 3, 91058 Erlangen, Germany

Stéphane Perrard†
Université PSL, ENS Paris, Département de Physique, LPENS. 24 rue Lhomond, 75005 Paris, France

Nicolas Vandewalle‡
GRASP, UR CESAM, Université de Liège, Allée du 6 aout 19, 4000 Liège, Belgium

Matthieu Labousse§
Gulliver, CNRS UMR 7083, ESPCI Paris et PSL Université, 10 rue Vauquelin, 75005 Paris, France
(Dated: July 25, 2022)

TABLE OF CONTENT

- Graf's decomposition theorem and intensity of the wave field
- Randomly created wave field
  - Average intensity in a random wave field
  - Average value of a_n^{rms} in the case of a randomly generated field
- Coupling between the particle and the waves
- Fitting parameters of the Gamma distribution.
- Link to the code
- Supplementary figures

GRAF'S DECOMPOSITION THEOREM AND INTENSITY OF THE WAVE FIELD

Let us consider an impact at coordinate $\mathbf{r}_j$ and study the resulting surface deformation at coordinate $\mathbf{r}$, as depicted in Fig.SI 7. For the sake of simplicity, the origin of the reference frame is set at the center of the harmonic potential. The vectors $\mathbf{r}$ and $\mathbf{r}_j$ define respectively the angles θ and θ_j with the x-axis. The interface elevation $\zeta_j(\mathbf{r})$ for infinite memory is given by

$$\zeta_j(\mathbf{r}) = \zeta_0 \mathrm{J}_0(k_F|\mathbf{r}-\mathbf{r}_j|). \tag{SI 1}$$

Graf's addition theorem [1] allows to express $\zeta_j(\mathbf{r})$ in terms of the distances $|\mathbf{r}|$ and $|\mathbf{r}_j|$ to the origin. The surface elevation for infinite memory writes

$$\zeta_j(\mathbf{r}) = \zeta_0 \sum_{p=-\infty}^{\infty} \mathrm{J}_p(k_F|\mathbf{r}|)\mathrm{J}_p(k_F|\mathbf{r}_j|)\exp\left(ip\left(\theta-\theta_j\right)\right), \tag{SI 2}$$

i being the imaginary unit. The functions J_p are the cylindrical Bessel functions of first kind and p-th order. For a collection of N impacts $\mathbf{r}_j$, the total wave field writes

$$\begin{aligned}\zeta(\mathbf{r}) &= \zeta_0 \sum_{j=1}^{N}\sum_{p=-\infty}^{\infty} \mathrm{J}_p(k_F|\mathbf{r}|)\mathrm{J}_p(k_F|\mathbf{r}_j|)\exp\left(ip\left(\theta-\theta_j\right)\right)\\ &= \sum_{p=-\infty}^{\infty} \underbrace{\zeta_0\left(\sum_{j=1}^{N}\mathrm{J}_p(k_F|\mathbf{r}_j|)\exp\left(-ip\theta_j\right)\right)}_{a_p}\mathrm{J}_p(k_F|\mathbf{r}|)\exp\left(ip\theta\right)\end{aligned} \tag{SI 3}$$

Note that, when considered, memory effects appear in the a_p coefficients. In this case, the amplitude writes

$$a_n = \zeta_0 \sum_{j=1}^{N}\mathrm{J}_p(k_F|\mathbf{r}_j|)\exp(-ip\theta_j)\exp\left(-\frac{j}{\mathrm{Me}}\right). \tag{SI 4}$$

Graf's addition theorem gives us a handy way to compute the arising wave field intensity. The intensity E is defined as follows

$$E = \lim_{R\to+\infty} \zeta_0^2 \frac{k_F}{2R}\int_0^R\int_0^{2\pi}\zeta(\mathbf{r})^2 r dr d\theta. \tag{SI 5}$$

Note that E is a dimensionless quantity related to the L-2 norm of the wave field. Strictly speaking, E is proportional to the sole gravitational energy of the fluid interface. The remaining capillary and kinetic energy contains terms involving the gradient of the fluid interface and the time derivative of the speed potential. This is why we take a special care to call E the wave intensity and not the wave energy.

The integral in Eq. (SI 5) does not converge, which explains why the limit is necessary for the definition. Indeed, as solution of the Helmholtz equation, $\mathrm{J}_p(x)$ scales as $x^{-1/2}$ because of energy conservation. More specifically, for large arguments, the Bessel functions can be approximated by

$$\mathrm{J}_p(x) \simeq \sqrt{\frac{2}{x\pi}}\cos\left(x-\frac{p\pi}{2}-\frac{\pi}{4}\right). \tag{SI 6}$$

Therefore, the integrand scales as $\mathrm{J}_p(k_F|\mathbf{r}|)^2 r \sim 1$ which leads to a divergent integral. One solution is to integrate up to a given radius R and to normalize the result by the diameter of the corresponding circle of integration as it has been done with Eq. (SI 5). By applying Parseval identity to Eq. (SI 5) in azimuthal directions we obtain

$$E = 2\pi \sum_{p=-\infty}^{\infty}|a_p|^2\left[\lim_{R\to+\infty}\frac{k_F}{2R}\int_0^R \mathrm{J}_p(k_F|\mathbf{r}|)^2 r dr\right]. \tag{SI 7}$$

To integrate over all positions, the limit $R \to \infty$ is used. This approach gives

$$\lim_{R\to\infty} \frac{1}{R}\int_0^R \mathrm{J}_p(k_F|\mathbf{r}|)^2 r dr = \frac{1}{\pi k_F}. \tag{SI 8}$$

This relation implies that all eigenmodes have the same energetic cost since the final result does not depend on p. Finally, the intensity writes

$$E = \sum_{p=-\infty}^{\infty} |a_p|^2. \tag{SI 9}$$

A last simplification can be made using Eq.(SI 3). The sum over p can be reduced to $p \in [0, \infty[$ instead of $p \in]-\infty, \infty[$ since a_p and a_{-p} share the same modulus. This yields

$$E = |a_0|^2 + 2\sum_{p=1}^{\infty} |a_p|^2. \tag{SI 10}$$

We have now an efficient way to measure the wave intensity as a superposition of central eigenmodes $\mathrm{J}_p(k_F|\mathbf{r}|)e^{ip\theta}$. All the a_p are stored in a double-infinite vector $\mathbf{a} = (\dots, a_{-p}, \dots, a_{-1}, a_0, a_1, \dots, a_p, \dots)$ which describes exactly the state of the interface at each instant. The modulus of this vector gives the wave intensity. In particular, the value of $|a_p|^2$ gives the energy stored in the eigenmode of index p.

RANDOMLY CREATED WAVE FIELD

Average energy in a random wave field

For the sake of simplicity, let us consider a 1D system where a collection of N sources $\{s^{(j)}\}$ is located randomly and uniformly in the interval $[0, \lambda_F]$. The wave field writes

$$\zeta(s, \{s^{(j)}\}) = \sum_{j=0}^{N-1} \underbrace{\zeta_0 \cos\left(\frac{2\pi}{\lambda_F}(s - s_j)\right) \exp\left(-\frac{j}{\mathrm{Me}}\right)}_{\zeta_j}. \tag{SI 11}$$

The average $\langle \zeta^2 \rangle$ is given by

$$\langle \zeta^2 \rangle = \frac{1}{\lambda_F^N} \int_0^{\lambda_F} \cdots \int_0^{\lambda_F} \zeta(s, \{s^{(j)}\})^2 ds^{(1)} \ldots ds^{(N)}, \tag{SI 12}$$

$$= \frac{1}{\lambda_F^N} \int_0^{\lambda_F} \cdots \int_0^{\lambda_F} \sum_{j=0}^{N-1} \sum_{k=0}^{N-1} \zeta_j \zeta_k \, ds^{(1)} \ldots ds^{(N)}. \tag{SI 13}$$

Since the impacts are uncorrelated, the product $\zeta_j \zeta_k$ gives the Dirac delta $\delta(j-k)$ which yields

$$\langle \zeta^2 \rangle = \frac{\zeta_0^2}{2} \sum_{j=0}^{N-1} \exp\left(-\frac{2j}{\mathrm{Me}}\right), \tag{SI 14}$$

$$= \frac{\zeta_0^2}{2} \frac{1 - \exp\left(-\frac{2N}{\mathrm{Me}}\right)}{1 - \exp\left(-\frac{2}{\mathrm{Me}}\right)}. \tag{SI 15}$$

Finally, assuming $N \to \infty$ and $\mathrm{Me} \gg 1$, one has

$$\langle \zeta^2 \rangle = \mathrm{Me} \frac{\zeta_0^2}{4} + \mathcal{O}\left(\mathrm{Me}^{-2}\right). \tag{SI 16}$$

Therefore, the wave intensity, which is proportional to $\langle \zeta^2 \rangle$, scales linearly with the memory.

Average value of a_n^{rms} in the case of a randomly generated field

In the case of a randomly generated wave field, one knows the radial distribution of impacts $\mathcal{P}(\mathbf{r})$ which is given by

$$\mathcal{P}(\mathbf{r}) = \left(\frac{1}{\sqrt{2\pi\sigma^2}}\right)^2 \exp\left(-\frac{|\mathbf{r}|^2}{2\sigma^2}\right). \tag{SI 17}$$

The angular distribution is taken as isotropic. Therefore the root mean squared value of a_n^{rms} is simply

$$(a^{\mathrm{rms}})_n^2 = \langle |a_n|^2 \rangle = \iint_{\mathbb{R}^2} a_n a_n^\dagger \mathcal{P}(\mathbf{r}) dx dy, \tag{SI 18}$$

$$= \frac{\zeta_0^2}{\sigma^2} \sum_{p=1}^{N} \exp\left(-\frac{2p}{\mathrm{Me}}\right) \int_0^\infty |\mathbf{r}| \left[\mathrm{J}_n\left(\frac{2\pi}{\lambda_F}|\mathbf{r}|\right)\right]^2 \exp\left(-\frac{|\mathbf{r}|^2}{2\sigma^2}\right) d|\mathbf{r}|, \tag{SI 19}$$

$$= \zeta_0^2 \frac{\mathrm{Me}}{2} \exp\left(-\frac{4\pi^2}{\lambda_F^2}\sigma^2\right) \mathrm{I}_n\left(\frac{4\pi^2}{\lambda_F^2}\sigma^2\right) \tag{SI 20}$$

where I_n is the modified Bessel of first kind of order n. To obtain the last equation, one has to use the relation regarding the integration of $x \exp\left(-p^2 x^2\right) \mathrm{J}_n(ax) \mathrm{J}_n(bx)$ [1].

COUPLING BETWEEN THE PARTICLE AND THE WAVES

In this section, we give an iterative map for the walking droplet dynamics that is encoded in the simulations. We first discuss the case of a flat surface without external forces in order to quantify the viscous dissipation in the dynamics. The viscous dissipation finds its origin in the lubrication effect that appears when the droplet sits on the surface, between two parabolic jumps. In the simulations, this dissipation is implemented via a linear damping factor resulting in an exponentially decreasing speed when the drop is in *contact* with the interface. The amplitude of the dissipation is determined by the time scale associated with the damping $\tau_v = 0.025$ s and the duration of the contact $\tau_c = 0.22\ \tau_F = 5.5$ ms. The value of this last parameter is set by the vibration parameters of the liquid surface. Including the external potentials is done by integrating the corresponding force on the elementary time step of the iterative equation during the parabolic flights of the drop of duration $\tau_f = 0.78\ T_F$.

$$\mathbf{v}_{i+1/2} = \exp\left(-\frac{\tau_c}{\tau_v}\right)\mathbf{v}_i - \frac{\tau_f}{m}\boldsymbol{\nabla}U(\mathbf{r}_i). \tag{SI 21}$$

$\mathbf{v}_{i+1/2}$ is the horizontal velocity just before the drop impact. The velocity after the impact is computed by adding interactions with the deformed surface is done as follow. The simulations assume that the impact with the liquid surface can be seen as inelastic, dissipating all the particle momentum perpendicular to the interface. We call the normal to the surface $\mathbf{N} = (\mathbf{n}, n_z)$ with $\mathbf{n} = (n_x, n_y)$ and the three-dimensional velocity $\mathbf{V} = (\mathbf{v}, v_z)$, with $\mathbf{v} = (v_x, v_y)$. Given the assumption of inelastic impact, the drop velocity after the impact writes

$$\mathbf{v}_{i+1} = v_{i+1/2} + |\mathbf{V}(t+1/2)\cdot\mathbf{N}|\mathbf{n} \tag{SI 22}$$

Note that $v_z(i+1/2)$ is a constant for a given acceleration amplitude coming from the parabolic flight (see [2] for the details). Mathematically,

$$\mathbf{N} = \frac{1}{\sqrt{1+\partial_x\zeta^2+\partial_y\zeta^2}}\left(-\partial_x\zeta, -\partial_y\zeta, 1\right). \tag{SI 23}$$

The iterative relation in the main text is recovered by remarking that $|\mathbf{V}.\mathbf{N}|\mathbf{n} \simeq v_z(i+1/2)\mathbf{n}$ and linearising $\mathbf{n} \simeq (-\partial_x\zeta, -\partial_y\zeta)$. The simulation keeps all the other non-linear terms that are called *higher order terms* in the main text.

FITTING PARAMETERS OF THE GAMMA DISTRIBUTION

The Gamma distribution used for the fitting of the PDF of the field intensity is done by considering the canonical form

$$P(x) = \frac{1}{c\Gamma(a)b^a}x^{a-1}e^{-\frac{x}{b}} \tag{SI 24}$$

with the fitting parameters

	Walker, 100 mHz	Walker, 250 mHz	Random, 100 mHz	Random, 250 mHz
a	12.101	18.456	41.61	17.84
b	197.421	60.194	58.48	141.86
c	100.806	49.447	18.50	19.50

LINK TO THE CODE

The algorithm can be found at the link https://mycore.core-cloud.net/index.php/s/bZsJUfvld9MxYbT

SUPPLEMENTARY FIGURES

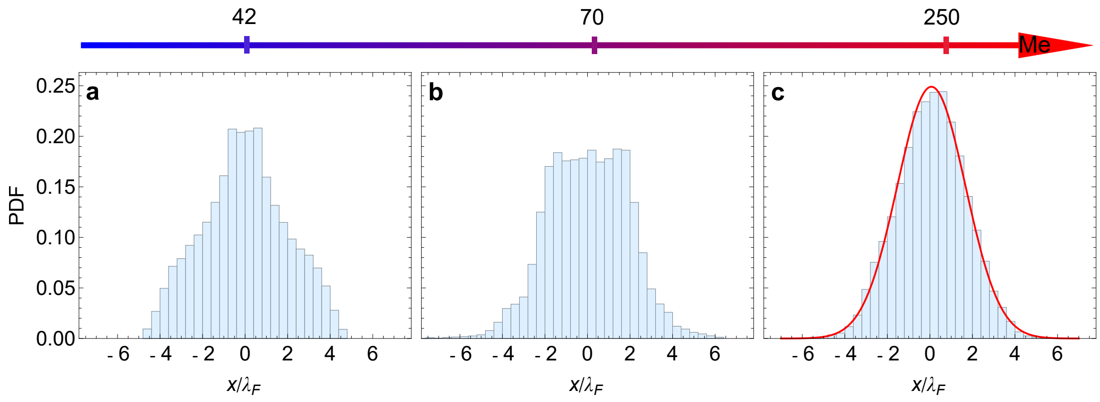

FIG. SI 1. Evolution of the positional statistics of the walker from the low memory regime to the high memory regime. Illustrations of the positional Probability Distribution Function (PDF) of the walker in numerical simulations, and associated to Fig 1d, 1e and 1f of the main article. **a** PDF for the position x of the walker at a memory parameter Me $= 42$ and frequency $\omega/2\pi = 0.250$ Hz. **b** PDF for the position x at a memory parameter Me $= 70$ and frequency $\omega/2\pi = 0.250$ Hz. **c** PDF for the position x at a memory parameter Me $= 250$ and frequency $\omega/2\pi = 0.250$ Hz. For all PDFs, 10^5 positions have been considered.

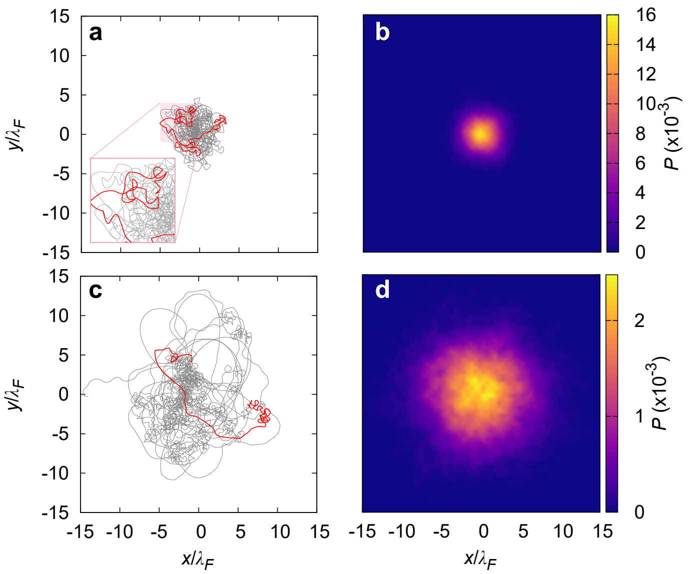

FIG. SI 2. Numerical dynamics of a walker confined in a harmonic potential in the high memory regime. **a** Trajectory of a walker and **b** the corresponding long-term probability distribution function P obtained in simulations, with a memory parameter Me $= 1\,000$ and a frequency $\omega/2\pi = 0.25$ Hz of the harmonic potential. **c** and **d** are similar to **(a)** and **(b)** but for $\omega/2\pi = 0.1$ Hz. The red lines on graphs **(a)** and **(c)** give a segment of the trajectory of duration τ, the memory time.

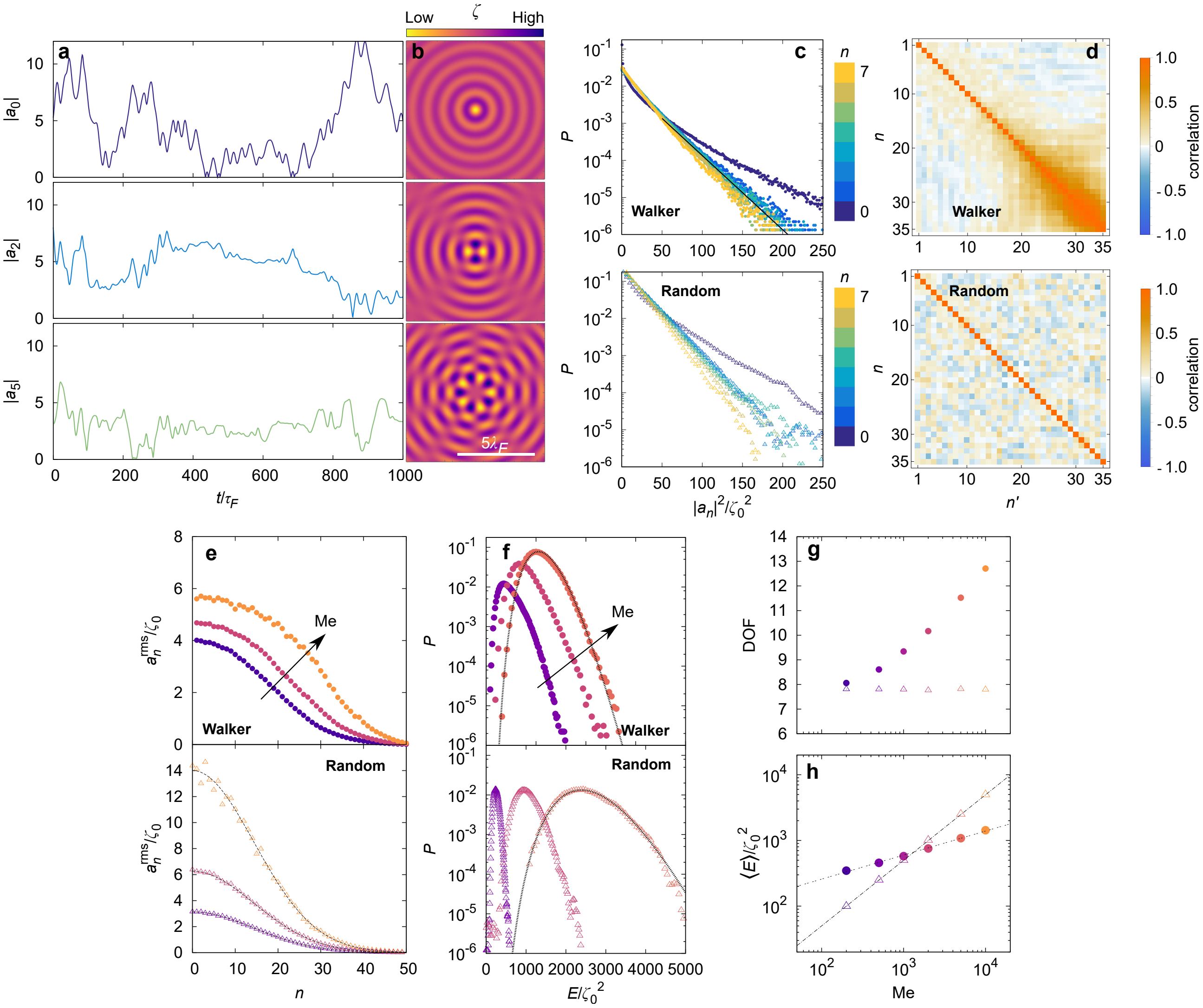

FIG. SI 3. Statistical description of the numerical dynamics of the wave field. This figure is the analogue of Fig.2 of the main text but for $\omega/2\pi = 0.10$ Hz. **a** Time series for the modulus of complex amplitude of the eigenmode n, $|a_n|$, for several different values of n, for a memory parameter Me $= 1\,000$ and frequency of the harmonic potential $\omega/2\pi = 0.10$ Hz, and **b** illustrations of the real part of the wave eigenmodes. **c** Probability density function P that the mode a_n takes the value $|a_n|^2$ for a walker (resp. randomly constructed field) for $n = 0, \ldots, 7$ (darkest color to lightest color) at a memory length Me $= 1\,000$ and a potential natural frequency of $\omega/(2\pi) = 0.10$ Hz (logarithmic scale along the y axis). **d** Correlation matrix for the modes a_n for $\omega/2\pi = 0.10$ Hz and Me $= 1\,000$. Both the random of the walker-generated and randomly-generated wavefield are presented. **e** Root-mean-squared (rms) value of a_n as a function of n for memory parameters Me $= 500, 2\,000$ and $10\,000$ (darkest color to lightest color) in the case of the walker dynamics (resp. randomly constructed field). **f** Probability density function for the wave intensity E (on logarithmic scale) for the walker dynamics (resp. randomly constructed field). Memory parameters are Me $= 500, 2\,000$ and $5\,000$. **g** Evolution of the number of degree of freedom DOF(see Eq.(6) of the main text) as a function of the memory parameter for the walker dynamics and the random dynamics on semi logarithmic scale along the x axis. **h** Comparison of the evolution of the average field intensity $\langle E\rangle$ with the memory parameters between the walker dynamics and the random dynamics on double logarithm scale. Dashed lines are power laws fitted on the simulation data. For the walker the exponent is obtained by fitting a power law and is 0.409 ± 0.027 (error is the 95%-confidence interval, coefficient of determination $R^2 = 0.9996$). For the random field the exponent is 0.992 ± 0.003 (errors account for a 95%-confidence interval, $R^2 = 0.999$).

* E-mail: maxime.hubert@fau.de
† E-mail: stephane.perrard@phys.ens.fr
‡ E-mail: nvandewalle@uliege.be

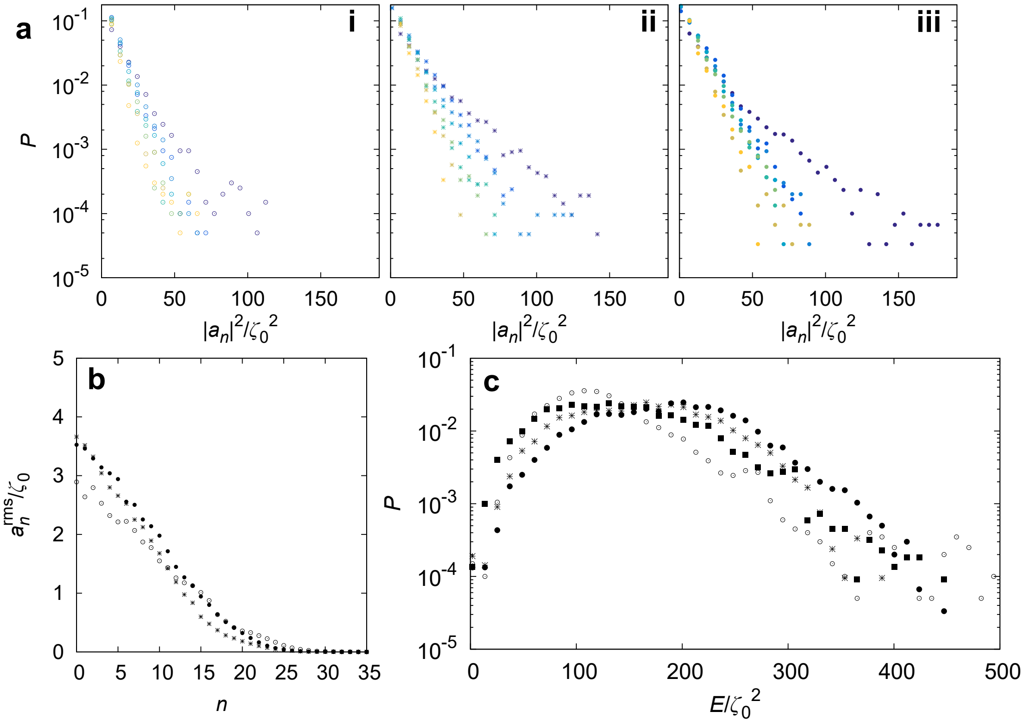

FIG. SI 4. Experimental measures related to the wavefield. The wavefield is reconstructed from the experimental paths and Eq. 2 of the main text. **a** Probability density functions P of the modulus of the complex amplitude, $|a_n|^2$, for index $n = 0, \ldots, 7$ (from blue to yellow). For (i, ii, iii) respectively: (frequency $\omega/2\pi$ (Hz), memory parameter Me): (0.36,250), (0.20,244), (0.30,244). **b** Root-mean-squared (rms) value of a_n, a_n^{rms}, and **c** probability density functions P of wavefield intensity E. In **(b)** and **(c)** the three curves correspond to the three different independent experiments in **(a)**. Black symbols: ($\omega/2\pi$ (Hz),Me): (0.36,250) ($\circ$), (0.20,244) ($*$), (0.30,244) ($\bullet$).

§ E-mail: matthieu.labousse@espci.psl.eu

[1] F. Olver, D. Lozier, B. R.F., and C. Clark, *NIST Handbook of Mathematical Functions* (Cambridge University Press, 2010).
[2] M. Labousse, *Etude d'une dynamique à mémoire de chemin: une expérimentation théorique*, Ph.D. thesis, Université Pierre et Marie Curie-Paris VI (2014).

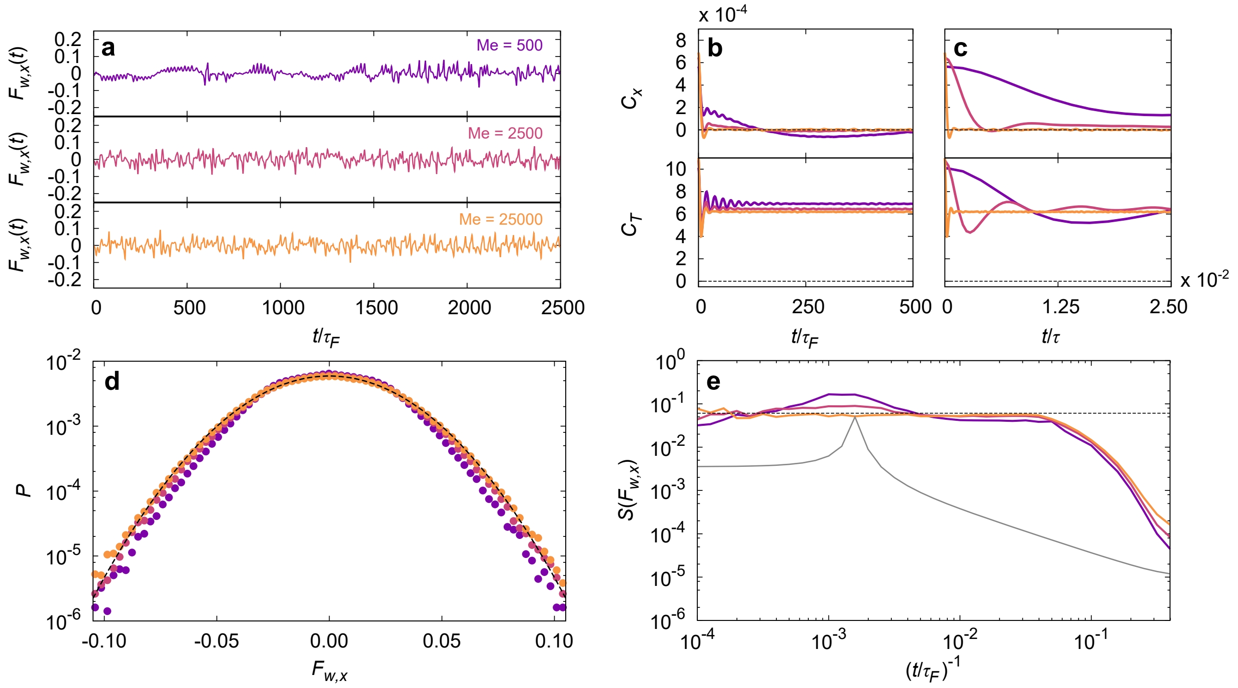

FIG. SI 5. The wavefield acts as a thermal reservoir for the walker. This figure is the analogue of Fig. 3 of the main text but for $\omega/2\pi = 0.10$ Hz. **a** Numerical time series of the wave force along the x direction, $F_{w,x}$, for different values of the memory parameter Me and the same frequency $\omega/2\pi = 0.10$ Hz. From top to bottom Me $= 500, 2\,500, 25\,000$. **b,c** Numerical correlation function for the wave force along the x direction, C_x, (top) and along the direction tangent to the velocity, C_T (bottom). The memory parameters and frequency are the same as in panel **(a)**. Two points of view are presented. The time t is rescaled by the bouncing time τ_F **(b)** and by the memory time τ **(c)**. **d** Numerical probability distribution function P for the wave force along the x direction on semi-logarithmic scale. Parameters are the same as in figure **(a)**. The solid gray curve is a Gaussian fit to the data obtained at Me $= 25\,000$. **e** Numerical density power spectrum S for the wave force along the x direction on double logarithmic scale. Parameters are the same as in figure **(a)**. The dashed black line is a guide to the eyes and the gray dashed line indicates the density power spectrum in the low memory parameter regime, i.e. Me $= 50$.

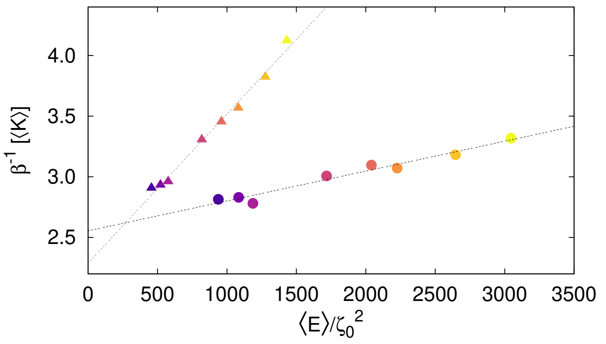

FIG. SI 6. Linear relation between the mean intensity stored in the memory field $\langle E\rangle$ and the walker effective temperature β^{-1} for a frequency $\omega/2\pi = 0.10$ Hz (circle) and $\omega/2\pi = 0.25$ Hz (triangle) and for memory parameters in the range [500 : 10000], shown as points from blue to green and of increasing brightness. The dashed lines are linear fits with equations $2.55 + 2.46 \times 10^{-4}\langle E\rangle$ (for $\omega/2\pi = 0.10$ Hz) and $2.29 + 12.3 \times 10^{-4}\langle E\rangle$ (for $\omega/2\pi = 0.25$ Hz) with a R^2 parameter of 0.963 and 0.989 respectively. Surprisingly, a linear relation is recovered for all points. Nevertheless, as expected, a direct proportionality between the two quantities is not recovered. We rationalize this by the fact that, at lower memory, the dynamics of the walker is not erratic and does not lead to a probability $\mathcal{P}(r)$ which follows a normal distribution. In this situation, the concept of temperature is ill-defined.

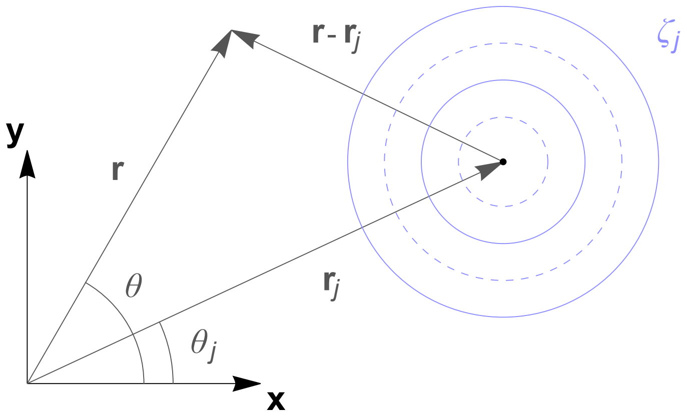

FIG. SI 7. Notations used for Graf's addition theorem in Eq. (SI 2). The wavefield at position $\mathbf{r}$ at orientation θ with respect of the $\mathbf{x}$ axis resulting from an impact at $\mathbf{r}_j$ at orientation θ_j with respect of the $\mathbf{x}$ is expressed in a basis of central Bessel function J_p.
